# International Migration and Development: A Dyadic Analysis of the Americas, 1970–2010

**DOI:** 10.3389/fsoc.2020.00015

**Published:** 2020-03-04

**Authors:** Matthew R. Sanderson, Jeffrey D. Kentor

**Affiliations:** ^1^Department of Sociology, Kansas State University, Manhattan, KS, United States; ^2^Department of Sociology, Wayne State University, Detroit, MI, United States

**Keywords:** migration, development, globalization, inequality, Americas

## Abstract

This paper assesses the migration-development nexus from a new, relational perspective, providing a closer test of existing theories of cross-national dynamics, including migration and development. Using bilateral data, we assess the relationship between migration (im)balances and wage differentials between pairs of countries in the Americas, from 1970 to 2010. The analysis reveals a positive feedback between international migration and cross-national inequalities. Migration responds strongly to wage gaps, which motivate more uni-directional, or imbalanced migration flows in country-pairs. This relationship is particularly strong in contiguous countries. Similarly, wage gaps respond to migration imbalances, which increase per capita income differences in country-pairs, although the effect of migration on wage differentials is smaller than the effect of wage differentials on migration. Together, the results suggest that the migration-development nexus is characterized by a strong internal momentum.

## Introduction

Migration is increasingly touted by key players in the policy realm as a means of addressing inequalities through “development.” As cross-national inequalities persist, questions are (re-) emerging about the relationship between migration, development, and inequality. How does immigration affect development outcomes in sending and receiving countries? Further, how do relative changes in cross-national inequalities affect the magnitude and timing of migration between countries? More broadly, how does international migration compare in magnitude to other, known global drivers of inequalities, including trade, foreign direct investment, and international political relations?

We assess these questions by taking a different approach, theoretically and methodologically. We integrate insights from neoclassical economics into a political-economic analytical framework that views both cross-national inequalities and international migration as expressions of an uneven distribution of power across countries situated within a single, worldwide economic division of labor (Portes, 1978; Sassen, 1988). We employ dyadic analysis (Krackhardt, 1988), which utilizes a dataset constructed with country-pairs rather than individual countries, using existing comprehensive cross-national, longitudinal data. Our approach offers a closer test of political-economic theory, which is oriented around *relational* explanations of cross-national dynamics, including migration and development. To begin, we construct a theoretical framework from neoclassical economic theory and political-economic theory, two approaches that have often been cast, for good reason, as counter-explanations of the migration-development nexus. As will be shown, however, these two perspectives are not entirely antithetical to one another on the issue of wage differentials, or gaps, which is the most important factor posited to shape migration patterns in the world division of labor.

## Conceptual Framework

Neoclassical economic theory takes as its point of departure the assumption that migrants are rational, utility-maximizing individuals who make the decision to move on the basis of cost-benefit considerations, the primary consideration being the expected wage gains from moving into the labor market in a destination country where labor is remunerated at a relatively higher level (Lewis, 1954; Lee, 1966). At the macro-level, international wage differentials are the key explanation of international migration (Thomas, 1973). International wage differentials are an outcome of differences in the relative supply of and demand for labor: wages will be lower in countries in which the supply of labor exceeds the demand for labor, and higher in countries in which the supply of labor is insufficient to meet demand. At the micro-level, migrants are posited to act rationally in exploiting differentials in international wage levels. Migrants are “pushed” by relatively lower wages in the origin country and “pulled” to relatively higher wages in the destination country. International migration should therefore reduce wage differentials between countries, moving countries closer to an equilibrium level that reflects only the costs of moving, and ultimately minimizing much of the economic incentive to move.

Although this perspective remains the conventional framework, it is increasingly criticized for its inability to account for empirical regularities. Most importantly, wage differentials have been shown to be only a weak explanation of international migration. Despite widening differences in cross-country wage levels, the propensity to send migrants varies dramatically across countries and the stock of international migrants has remained relatively stable at three per cent of world population since the 1960s. Moreover, wages across countries have not come close to converging, despite the fact that there are more international migrants today in absolute numbers (232 million) than at any time in modern history (United Nations, 2013).

The limitations of the conventional perspective opened intellectual space for alternative perspectives, including political-economic approaches. In contrast to the neoclassical economics concept of rational, utility-maximizing migrants, political economy approaches focus on the structures that condition and constrain individual action. Migration is part of a system: individuals may indeed migrate on the basis of cost-benefit considerations, but both the costs and benefits of movement are structured by an historical context of unequal exchange in a hierarchical international division of labor. By definition, international migration involves the transgression of national boundaries. But for those working from a political economy perspective, migration is not only movement across national boundaries; it is more importantly movement *within* an integrated political-economic system (Portes, 1978; Sassen-Koob, 1978, 1981; Portes and Walton, 1981; Delgado Wise, 2006; Delgado Wise and Covarrubias, 2007, 2008; Delgado Wise and Cypher, 2007).

Placing international migration in this broader, world-historical context addresses a key limitation in the conventional, push-pull framework: that cross-country wage differentials are not strong explanations of international migrations. By expanding the scope of inquiry from national to world-scale processes, the political economy perspective opens up for examination the relationship between trans-national political-economic processes and international migration (Petras, 1980; Morawska, 1990; Hamilton and Chinchilla, 1991, 1996; Fernandez-Kelly and Massey, 2007). This is an important analytical advance because these global processes ultimately create the context for individual-level decision-making: “It is within the context of extensive social and economic penetration of peripheral societies by the institutions of advanced capitalism that individual cost-benefit calculations make sense” (Portes, 2007, p. 77). In this sense: “Migrants can be viewed as stepping or falling into a migratory flow, rather than initiating or constituting such a flow through their individual decisions and actions” (Sassen-Koob, 1978, p. 515).

By focusing almost exclusively on wage differentials, the conventional perspective also de-politicizes the world political-economic context in which migrants make decisions. In doing so, it misses key, structural relations that motivate and sustain international migration. Here, the concept of unequal exchange is paramount. Over time, unequal exchange between core and non-core zones produces uneven development across zones in the world-economy. International migration is a *consequence* of this uneven development, expressed as wage differentials: if not for (widening) cross-national income disparities, international migration would not exist. In this sense, both the conventional approach and the political economy perspective view wage differentials as a necessary cause of international migration.

### Migration as a Cause of Wage Differentials and Unequal Exchange

Although both approaches view wage differentials as motivations for migration, these differentials become an explanandum in the political economy approach; they are foregrounded and explained, whereas they remain exogenous to the conventional approach.

Political economists contend that international migration is not only an outcome of unequal exchange, it is also *cause of* unequal exchange, reproducing uneven development in the world political economy. Burawoy (1976) provides one of the earliest discussions. International migration is a labor supply system. Migrants are a labor force, and like all labor forces, it must be maintained and renewed, or reproduced. What differentiates an international labor supply system from a domestic one, however, is that the process of reproduction (that is, of maintenance and renewal) occurs *across* national boundaries, so that different institutions are responsible for organizing, and bearing the costs of, the reproduction of the labor force. This opens up the possibility that the benefits of migrant labor may not accrue to the institutions bearing the costs of reproducing the migrant labor system. For example, in the case of Mexican emigration to the United States, the costs of educating, training and reproducing the labor force are borne largely by the Mexican state and economy, but the benefits of capital accumulation derived from their application to production processes are reaped mainly by the United States:

“Thus, for Mexican migrants, processes of renewal are organized under the Mexican state in the Mexican economy, and those of maintenance in the United States…the activities of maintenance and renewal are separated…In other words, a proportion of the costs of renewal is externalized to an alternate economy and/or state” (Burawoy, 1976: 1052-1053).

Thus, international migration is a form of unequal exchange, reproducing uneven development: “The significance of migrant labor lies in the separation of the processes of maintenance and renewal, so that renewal takes place where living standards are low and maintenance takes place within easy access of employment” (Burawoy, 1976, p. 1082). By capitalizing on uneven levels of wage remuneration across countries, international migration then tends to exacerbate those differences, leaving the origin country with sunk costs associated with education, training, and reproducing labor while enhancing capital accumulation in the destination country: “The very sale of labor power by an underdeveloped country…to an economically advanced nation serves only to reinforce the relations of economic subjugation and domination” (Burawoy, 1976, p. 1068).

Understanding international migration as a cross-national labor supply system means understanding migration as an unequal exchange between nation-states *within* a hierarchical world political economy and not as isolated movements across autonomous, self-contained nation-states (Sassen, 1988, 2001). That is, international migration is inherently *relational*—it is an exchange between two countries. From the political economy perspective, this exchange is both an outcome of uneven development (expressed as wage differentials) and a cause of uneven development between two countries.

### Migration and Structural Imbalances

As an exchange, international migration is closely associated with structural imbalances within the origin and destination countries. Power differences between origin and destination countries, expressed as per capita income differentials, cannot be ignored. International migration is both an outcome of cross-national power differentials and is a contributor to them.

From the political economy perspective, international migration is initiated as higher-income countries expand markets into, or penetrate, lower-income countries. Market expansion through trade and foreign direct investment (FDI) restructures social, political, economic, and cultural institutions, mobilizing segments of the population into migration streams (Sassen, 1988), some of which are directed toward domestic urban areas and some of which spills over across national boundaries: “Sustained labor migration requires the penetration of the political and economic institutions of the dominant unit…into the subordinate one…(creating) internal imbalances between sectors and institutions in the subordinated unit” (Portes and Walton, 1981, p. 31).

Integration between higher and lower-income countries creates bi-national markets for labor and capital that motivate migration. As institutions within the lower-income country are restructured to fit into the bi-national, and inter-national, division of labor, new domestic classes emerge with closer ties to foreign capital, and consumption habits, and values and norms are reoriented toward the higher-income country. In particular, the balance between labor and capital within the origin and destination countries changes. On the labor-supply side (in the origin country), newly mobile populations emerge as labor is “freed up” from traditional sectors such as agriculture. As a result, international migrations do not originate “spontaneously” from individual cost-benefit analyses. They are produced by political-economic processes that imbalance the lower-income society in relation to the higher-income country “Structural imbalances between newer and older elements eventually produce migratory pressures” (Portes and Walton, 1981, p. 32). The concept of structural imbalancing is supported with case studies that range from South African manual labor migrations to the emigration of Argentine doctors, providing empirical evidence that “…common forces underlie superficially different movements” (Portes and Walton, 1981, p. 30).

On the labor-demand side (destination country), supply-side shocks induce new demands for lower-wage labor that support further capital accumulation in the higher-income country and exacerbate wage differentials between the origin and destination country. International migration is thus motivated by this “pull”-effect of restructuring while promoting further economic restructuring in the higher-income country (Piore, 1979). Here, the value of a political-economic perspective is particularly apparent, as these two dynamics—on the supply and demand side—are viewed as flip-sides of a *single* bi-national, or international, process: the restructuring of capital accumulation beginning in the 1960s. Deindustrialization in the core and the restructuring of core economies into service-oriented economies increased demand for both high-wage *and* low-wage service sector jobs, polarizing occupational and income distributions and increasing the demand for immigrant labor (Piore, 1979). Motivated by the need to sustain profitability in the face of rising wages in the core, corporations in high-income countries invest in production abroad, expanding markets, and this investment ultimately mobilizes segments of lower-income countries into migration streams that are directed back toward the high-income country (Sassen, 1988).

Political economy approaches thus explicitly relate wage differentials to international migration in a reciprocal relationship. Migration is an exchange. On the one hand, wage differentials express inequalities in power between two countries. Higher-income countries are able to re-structure institutions in lower-income countries, leading to structural imbalances that give rise to international migration. On the other hand, international migration promotes further structural imbalances in both origin and destination, exacerbating wage differentials between high and lower-income countries.

Thus, from the political economy perspective, there are not one, but two indicators of unequal exchange—income differentials *and* migration differentials—*and they are related*. Income differentials should motivate international migration between the two countries, resulting in a migration *imbalance*—a larger flow of immigrants moving in one direction—between the two countries. Further, this migration imbalance should exacerbate wage differentials between the two countries, as it would facilitate restructuring within the higher-income country.

We test these relationships using bilateral data on country-pairs. In doing so, we extend the concept of structural imbalancing beyond a particular country to bi-national contexts. Here, structural imbalancing is relational—it occurs within the context of exchanges between countries, of which migration is one such key exchange. This approach provides a closer test of political economy frameworks, as it is able to assess relations between origin and destination countries simultaneously. We move beyond the country characteristics approach to migration and development and recast this relationship more clearly in a dyadic, relational perspective.

As a further extension, we assess the role of geography as a moderating factor. Although it has diversified geographically, international migration remains more common between countries that are contiguous, especially in the Global South (Ratha and William, 2007). Migration is both a cause and a consequence of labor markets that form across national boundaries. For example, Sanderson (2014) found that movements of capital and labor between Mexico and the U.S. created a bi-national labor market linking the two countries. Capital investments between the two countries created “channels” for migration that facilitated movement along sectoral-industrial lines between the two countries. The outcome of capital and labor movements between the two country was effectively a bi-national market for labor. We therefore explore the role of labor market contiguity as a possible moderating factor on the migration-development nexus.

## Data and Methods

### Dyadic Analysis

This study utilizes a dyadic analytic model to assess the relationships between migration and development, rather than the typical individual country attributional analytic structure. Dyadic analysis can more rigorously test existing theory, which posits that *relations between* particular countries shape development dynamics (Krackhardt, 1988; William, 2001).

Since the use of dyadic analysis in this type of research is relatively new, it may be useful to provide some background on this methodology. The use of dyads is an old concept, originating in psychology nearly a century ago with the study of pairs of individuals as the unit of analysis (Picard, 1920). It first appears in the sociological literature in the early 1940s (Becker and Useem, 1942), again with pairs of individuals as dyads.

The field of international relations has employed this methodology in studies of between-country relationships extensively (see Erikson et al., 2017). However, the use of dyads in the sociological literature with country-pairs as the unit of analysis emerged only recently (see, for example, Bonikowski, 2010), and the use of dyadic analysis in the study of migration is even more recent (Blodgett and Leblang, 2015).

### Dyadic Data Structure

Our dyadic data set is considerably more complex than an individual country-based structure. A typical attributional data set for the Americas would have 22 countries, or cases. In our dyadic data structure, the unit of analysis is the country-pair (U.S. Mexico, for example), giving us 462 total country-pairs (22 × 21 = 462). Half of these pairs are redundant (Honduras-Brazil and Brazil-Honduras, for example), leaving 231 independent country-pairs. Since our study is longitudinal, with five time points (1970, 1980, 1990, 2000, 2010), the individual case becomes the country-pair-year (i.e., Belize-Columbia-2010). Our final data set contains a total of 1,155 country-pair-year cases (or dyad-year cases; 231 × 5 = 1,155).

Given the longitudinal structure of our analyses, we estimate models using the two most common panel data methods—random effects and fixed effects models—to address the problem of unobserved heterogeneity. We use random effects models specifically to assess the influence of geographic contiguity, or a shared border. Because contiguity is a time-constant, unit-specific variable, it is effectively removed from fixed effects analysis, making it only possible to analyze in random effects models.

Data for this study come from several different cross-national data sources. Bilateral international migration stocks and bilateral refugee stocks come from the World Bank's Global Bilateral Migration Database (2014). To ensure that migration stocks do not include refugee stocks, we subtract the refugee stocks from migration stocks for each country pair. We use stocks for theoretical and methodological reasons. Theoretically, both political economy and neoclassical economic approaches make arguments about total numbers of immigrants, so we use total numbers, or stocks, of immigrants as our measure of migration, and control for the country's population size in the analyses. Methodologically, bilateral data on migration flows were not available in sufficient numbers for analysis. Additionally, using migrant stocks can be seen as a more conservative approach, because stocks are relatively more stable than flows over time^1^.

Included in our analyses are several control variables used in previous research. *GDP per capita* data are in constant 2000 U.S. dollars and are taken from the World Bank's World Development Indicators dataset (2014). *Total population, government expenditures per GDP* and *foreign direct investment (FDI) stocks per GDP* are also taken from the WDI dataset.^2^ *Income inequality* data are from the Standardized World Income Inequality Database (SWIID) (version 15), which provides comparable GINI indices of net income inequality. *International Governmental Organizations* (IGO) data and *bilateral imports* data were produced by the Correlates of War project (version 2.1). We standardize the imports data on GDP for purposes of comparison. In addition, we include two interaction terms of border contiguity^*^international migration and border contiguity^*^GDP per capita.

Because we are interested in how *differences in magnitudes between* countries affect international migration and income differences, we compute difference scores for all of the variables. For example, to determine the “wage gap” between two countries in given year, we use the absolute value of the difference between the GDP per capita of Country A and the GDP per capita of Country B. Similarly, to determine the “migration balance/gap,” we use the absolute value of the difference between the number of international migrants from Country A living in Country B, and the number of international migrants from Country B living in Country A. For example, if there are 8,686 Americans in Argentina in 1970 and 55,325 Argentinians in the U.S., then the migration gap, or balance, for this dyad-year is 46,639. We use the absolute value of this difference so that the measure does not depend on the direction of the difference (i.e., whether the value for Country A is subtracted from the value of Country B, or vice versa). This strategy allows us to more closely test political-economic theory, which is a *relational* approach focused on the imbalances, or *differences the in magnitudes* of wages and migration between countries.

Sample sizes are determined by data availability. The reduced models (i.e., models without controls) include all (100%) possible dyads (*n* = 231) and 92% of all possible *dyad-years* (*n* = 1,077). Data are only available from 1980 to 2000 for all the variables in the full model, reducing the total number of dyads in the analysis to 209 (91% of all possible dyads) and the number of *dyad-years* to 354 (31% of all possible dyad-years).

## Results

Table 1 presents results from the analysis of migration balances (gaps). Consistent with political economy theory and neoclassical economic theory, wage differentials are positively associated with migration balances. Migration stocks are more imbalanced in country-pairs with larger wage gaps. The effect of wage differentials depends in part, however, on contiguity (Model 3 in Table 1). The positive effect of wage differentials is much larger in country-pairs that share a border. The main effect for GDP per capita indicates that for country-pairs that do not share a border, a 10% increase in the wage gap is associated with a 5.2% increase in the migration imbalance (1.10∧0.533 = 1.052). However, for country-pairs that share a border, a 10% increase in the wage gap is associated with a 69.2% increase in the migration imbalance (5.895–0.376 = 5.519; 1.10∧5.519 = 1.692). Thus, as neoclassical economic theory and political economy theories expect, wage differentials give rise to more uni-directional flows of immigrants between countries. Moreover, this effect is much stronger in country-pairs that are contiguous.

**Table 1:** Dyadic panel regressions of international migration gaps on GDP per capita gaps.

	**Model 1**	**Model 2**	**Model 3**
	**REM**	**FEM**	**REM**
Difference in level of development [GDP per capita]	0.331^***^	0.153^*^	0.533^***^
	5.02	2.49	4.51
Contiguity [Shared border = 1]			5.895^***^
			4.55
Border^*^GDPpc			−0.376^*^
			−2.07
Difference in Trade [Imports per GDP]			0.146^***^
			3.82
Difference in income inequality [GINI]			0.334^*^
			2.29
Difference in total populations			0.0842
			0.84
Difference in international governmental organization memberships [IGOs]			−1.154
			−1.85
Difference in foreign direct investment levels [FDI stock per GDP]			0.261^**^
			3.12
Difference in government state strength [Government expenditures per GDP]			−0.394^***^
			−3.41
1980	0.222^**^	0.276^***^	
	2.71	3.42	
1990	0.281^**^	0.338^**^	0.257
	2.72	3.26	0.77
2000	0.592^***^	0.687^***^	0.056
	5.75	6.49	0.16
2010	1.036^***^	1.135^***^	
	8.03	8.69	
Constant	3.353^***^	4.845^***^	2.116
	6.4	10.27	0.55
N [dyad-years]	1,077	1,077	354
N [dyads]	231	231	209
R^2^ (overall)	0.204	0.134	0.603

*t-statistics in parentheses*.

* *p < 0.05*,

**\ *p < 0.01*,

*** *p < 0.001 (two-tailed tests)*.

We also note that both other measures of globalization—trade and FDI—have positive relationships with migration balances. That is, migration becomes more imbalanced as trade gaps and investment (FDI) gaps widen. The coefficient for the difference in within-country inequality is also noteworthy. As the difference in within-country inequality scores increases, migration imbalances increase, too. We note that government expenditures, a measure of state strength, are the only variable negatively associated with migration gaps. We find that migration balances decrease, or become more even, in country-pairs as the difference between state strength increases. Finally, migration gaps are growing over time, as indicated by each of the time trend variables.

Table 2 presents results from the analysis of wage differentials (gaps). The findings lend support to political economic theory. Migration imbalances are associated with higher wage differentials, not lower wage differentials as neoclassical economic theory would expect. Larger migration imbalances in country-pairs are associated with larger wage gaps. This relationship does not depend on contiguity, as the interaction term is not statistically significant. The main effect for international migration indicates that a 10% increase in the migration imbalance (gap) is associated with a 1.3% increase in the wage gap (1.10∧0.135 = 1.0129).

**Table 2:** Dyadic panel regressions of GDP per capita gaps on international migration gaps.

	**Model 4**	**Model 5**	**Model 6**
	**REM**	**FEM**	**REM**
Difference in international migration [Total stock of immigrants from partner country]	0.124^***^	0.0589^*^	0.135^***^
	(6.36)	(2.47)	(4.77)
Contiguity [Shared border = 1]			−0.975
			(−1.75)
Border^*^Int'l Migration			0.000691
			(0.01)
Difference in Trade [Imports per GDP]			−0.0232
			(−1.23)
Difference in income inequality [GINI]			0.262^***^
			(3.70)
Difference in total populations			−0.0782
			(−1.18)
Difference in international governmental organization memberships [IGOs]			−0.737^**^
			(−3.00)
Difference in foreign direct investment levels [FDI stock per GDP]			0.327^***^
			(5.82)
Difference in government state strength [Government expenditures per GDP]			0.113
			(1.93)
1980	0.155^*^	0.175^**^	
	(2.51)	(2.80)	
1990	0.165^*^	0.189^**^	−0.586^***^
	(2.33)	(2.66)	(−4.80)
2000	0.332^***^	0.381^***^	−0.469^***^
	(4.26)	(4.69)	(−3.85)
2010	0.537^***^	0.613^***^	
	(6.11)	(6.85)	
Constant	6.701^***^	7.102^***^	9.515^***^
	(54.36)	(51.95)	(5.67)
N [dyad-years]	1,077	1,077	354
N [dyads]	231	231	209
R^2^ (overall)	0.213	0.163	0.609

*** p < 0.001;

** p < 0.01;

* *p < 0.05*.

Differences in foreign direct investment (FDI) and within-country income inequality also are positively associated with wage gaps. Stated differently, more imbalanced FDI stocks and larger differences in within-country income inequality are associated with larger wage differentials between countries. We also note that the year indicators demonstrate growing wage gaps in the region from 1970 to 2010.

## Discussion

This paper assessed the migration-development nexus from a new, relational perspective, using bilateral data to assess the relationship between migration balances and wage differentials between pairs of countries in the Americas, from 1970 to 2010. The findings have important implications for: our understanding of the links between the migration, development, and inequalities; and theory and policy related to the migration-inequality-development nexus, which now stands as a top priority in the global development agenda among key international organizations.

The most important finding to emerge is the existence of a positive feedback between international migration and cross-national inequalities. In line with neoclassical economic theory and political economic theory, wage differentials motivate international migration, which manifest as migration *im*balances in country pairs. But, consistent with political-economic theory, international migration imbalances lead to *larger* wage differentials in country-pairs. Thus, the results suggest a significant internal momentum in the migration-development nexus. Migration responds to cross-national inequalities (wage gaps), and as migrants become more concentrated in higher-income countries, wage gaps increase, which motivates further migration in a positive feedback loop that exacerbates cross-national inequalities.

The strength of the relationship depends on geographic contiguity. Wage gaps are positively associated with migration imbalances regardless of whether countries share a border, but the effect of wage differentials is highly elastic in contiguous counties. If average incomes in a county-pair sharing a border diverge by 1%, the migration balance would become much more uneven, increasing by ~6%. To translate this in to real terms, the wage differential between the U.S. and Mexico in 2010 was $35,835 (absolute value of $43,952–$8,117 in 2000 constant U.S. dollars) and the migration balance was 11,757,661 (absolute value of 740,182–12,497,843). Every 1% increase in the wage gap ($358), is associated with an increase in the migration imbalance of 658,529 persons.

Although there is a positive feedback between wage gaps and migration balances, the two relationships are not proportional, and the result is a more muted feedback than would otherwise be the case. Stated differently, migration responds strongly to wage gaps, but wage gaps are not as responsive to migration. Increasing the migration imbalance by 10% would lead to just a 1.3% increase in the wage gap. It is a significant response, but it is much weaker than the migration response to a change in the wage differential. In this regard, one finding merits more attention in future research. We find that the wage differential-migration balance relationship is much more elastic than the migration balance-wage differential relationship. The difference between the magnitude of the effects is intriguing and is worthy of further exploration.

This study provides a basis for further theoretical integration, as we find evidence to support both neoclassical economic theory and political economy theories. New data available at the bilateral level of analysis should make integration much more feasible in the coming years. It is noted that the U.S.-Mexico relationship is important for understanding migration and development in the western hemisphere. The Mexico-U.S. migration corridor is the largest in the world in terms of the number of migrants moving between these two countries. Future research could expand the scope beyond the Americas in order to examine the generalizability of the findings. It would be worthwhile to assess other regions specifically in order to understand whether the dynamics we have identified in the U.S./North American-based migration system generalize to other world regions. Similarly, there is a real need to incorporate measures of conflict. Data on conflict (internal and international) are readily available. In the Americas, there was insufficient variation to warrant inclusion of conflict data in our models. Moreover, our models remove refugee stocks from the migration stocks variable. However, the role of conflict on the migration-development nexus should be considered in future research examining a wider array of countries and/or different world regions. Finally, the significant impacts we find of income inequality, foreign direct investment, IGOs and trade on the migration/wage nexus all offer fertile directions for future exploration.

## Data Availability Statement

Publicly available datasets were analyzed in this study. These data can be found here: https://datacatalog.worldbank.org/dataset/global-bilateral-migration-database; https://datacatalog.worldbank.org/dataset/global-bilateral-migration-database/a.

## Author Contributions

MS and JK contributed equally to authorship of the manuscript, including research design, conducting the research, performing the analysis, and writing the manuscript.

### Conflict of Interest

The authors declare that the research was conducted in the absence of any commercial or financial relationships that could be construed as a potential conflict of interest.

## Footnotes

^1^To further explore the relationship between migration balances and wage gaps, the models were re-estimated using international migration densities, or international immigrants per capita, instead of total international migration stocks. We find no evidence of a relationship between migration balances and wage gaps using international migration densities. Results are available upon request.

^2^Attributional FDI data are used in these analyses due to a lack of available bilateral FDI data.
